# Surgical treatment of diabetic foot: a spatial analysis, Ceará, Brazil, 2016-2021

**DOI:** 10.1590/S2237-96222026v35e20240755.en

**Published:** 2025-12-19

**Authors:** Yterfania Soares Feitosa, Bruno Victor Barros Cabral, Gabriela de Sousa Lima, Emiliana Bezerra Gomes, George Jó Bezerra Sousa, Thereza Maria Magalhães Moreira, Maria Lúcia Duarte Pereira

**Affiliations:** 1 Universidade Estadual do Ceará, Programa de Pós-Graduação em Cuidados Clínicos em Enfermagem e Saúde, Fortaleza, CE, Brazil; 2 Universidade Regional do Cariri, Enfermagem, Crato, CE, Brazil; 3 Ministério da Saúde, Brasília, DF, Brazil

**Keywords:** Diabetes, Diabetic Foot, Amputation, Surgical, Debridement, Observational Studies, Diabetes, Pie Diabético, Amputación Quirúrgica, Desbridamiento, Estudios Observacionales

## Abstract

**Objective:**

To identify the spatial pattern of surgical treatment of diabetic foot complications in Ceará.

**Methods:**

This was an observational spatial analysis study conducted in Ceará. We calculated the annual incidence rate of diabetic foot surgery. This rate was standardized with the indirect method using the mean year (2018). Subsequently, the rate was smoothed using the local empirical Bayes method. Furthermore, the Getis-Ord Gi* technique was applied to identify clustered areas of high incidence. Finally, a purely spatial scan was performed to identify relative risk (RR) for each municipality in Ceará, with those with a RR>1 presenting a higher risk for the event of interest.

**Results:**

Between 2016 and 2021, 7,319 hospitalizations for surgical treatment of complicated diabetic foot were recorded in Ceará, especially in the Fortaleza macro-region (49.8%; n=3,650). In the spatial analysis, both the crude rates, as well as the rates after local empirical Bayes smoothing, show greater concentration in the following macro-regions: Fortaleza, Sertão Central, Sobral and Cariri. This information was confirmed by local Moran’s I and the Getis-Ord Gi* statistic (p-value <0.05). The scan revealed that 30.5% (n=56) of the state’s municipalities presented RR>1.

**Conclusion:**

The study revealed a spatial pattern marked by concentration of diabetic foot surgical treatment cases in the Fortaleza macro-region and, after Bayes smoothing, revealed a shift in the distribution of cases to two of the state’s interior-region municipalities, Quixadá and Aracoiaba.

## Resumen

**Objetivo:**

Identificar el patrón espacial del tratamiento quirúrgico de las complicaciones del pie diabético en Ceará.

**Métodos:**

Estudio observacional de análisis espacial realizado en Ceará. Para el estudio, se calculó la tasa de incidencia anual de cirugía de pie diabético. Esta tasa se estandarizó mediante el método indirecto, utilizando la media anual (2018). Posteriormente, la tasa se suavizó mediante el método bayesiano empírico local. Además, se aplicó la técnica Getis-Ord Gi* para identificar áreas agrupadas de alta incidencia. Finalmente, se realizó un análisis espacial puro para identificar el riesgo relativo (RR) de cada municipio de Ceará; aquellos con un RR > 1 presentaron un mayor riesgo de presentar el evento de interés.

**Resultados:**

Entre 2016 y 2021, se registraron 7.319 hospitalizaciones por tratamiento quirúrgico de pie diabético complicado en Ceará, con destaque en la macrorregión de Fortaleza (49,8%; n=3.650). En el análisis espacial, las tasas brutas, así como las tasas tras el suavizado bayesiano empírico local, muestran una mayor concentración en las siguientes macrorregiones: Fortaleza, Sertão Central, Sobral y Cariri. Esta información se confirmó mediante el I de Moran local y el Getis-Ord Gi* (valor p < 0,05). En el análisis, se observó que el 30,5 % (n=56) de los municipios del estado presentó un RR> 1.

**Conclusión:**

El estudio reveló un patrón espacial marcado por la concentración de casos de tratamiento quirúrgico de pie diabético en la macrorregión de Fortaleza y, tras el suavizado bayesiano, reveló un desplazamiento en la distribución de casos hacia dos municipios del interior, Quixadá y Aracoiaba.

**Ethical aspects**

This research used public domain anonymized databases.

## Introduction 

By 2045, prevalence of type 2 diabetes is expected to increase by 21.1% in underdeveloped countries, 12.2% in developed countries and 11.9% in low-income countries (1). Foot ulcers stand out among the complications associated with the disease, which are of particular concern due to their significant impact on morbidity and mortality, in addition to causing disabilities (2).

Serious complications associated with diabetes, such as foot ulcers, when not diagnosed and treated early, can progress to infections and lower limb amputations. These complications increase mortality rates and result in physical disability, reduced work activity, increased health care costs and social and emotional harm (2).

In 2022, 18,811 amputation cases were recorded in Brazil, of which 952 occurred in the state of Ceará, representing an increase of 244.9% compared to 2013. Ceará came in 17^th^ place in the national ranking, while it was in 3^rd^ place in the Northeast region of the country, with a rate of 10.9 amputations per 100,000 inhabitants. The Cariri region, in Ceará, had the highest rate, namely 13.7 amputations per 100,000 inhabitants (3).

In this context, analyzing the spatial distribution of lower limb amputations is a crucial strategy for understanding geographic variations and identifying areas of high prevalence. Their autocorrelation allows us to detect significant geographic patterns that may indicate concentration of cases in certain regions, potentially guiding health care planning.

Exploring these spatial patterns is essential for uncovering factors underlying the concentration of cases in territories over time and for identifying possible causes or social determinants, such as socioeconomic inequalities, barriers to access to health services and environmental variables that influence the occurrence of these amputations (4).

Devising actions that optimize the allocation of resources for health promotion and disease prevention in regions with the highest number of amputations would certainly impact the quality of life of people with diabetes and foot complications, as well as reducing the social and economic costs for these individuals and for the Brazilian National Health System. Therefore, the objective of this study is to identify the spatial pattern of diabetic foot surgery in the state of Ceará, Brazil.

## Methods 

### Design 

This is an observational spatial analysis study conducted in Ceará, taking all 184 municipalities that make up the state as the unit of analysis. The study followed the recommendations of the Strengthening the Reporting of Observational Studies in Epidemiology (STROBE) Statement (5). 

### Setting 

**Figure 1:**
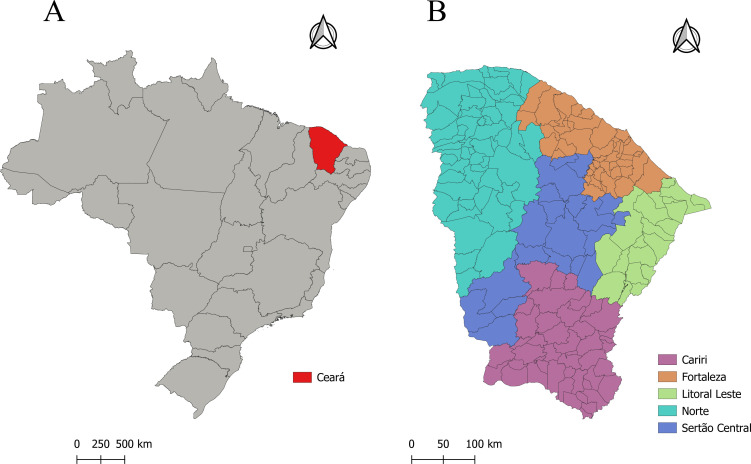
Geographic representation of the state in Brazil (A) and its division into five health macro-regions (B). Ceará, 2025

Ceará is divided into five health macro-regions: Litoral Leste, Sertão Central, Cariri, Sobral and Fortaleza, with a territorial extension of 148,894.447 km^2^ and an estimated population of 8,794,957 people (Figure 1). This study used data from the Hospital Information System, accessed by the Information Technology Department of the Brazilian National Health System, on cases of surgical treatment of diabetic foot (revascularization, debridement and amputation) (3) related to lower limb complications in people with diabetes (6).

We also used information on the population with diabetes in the state, held on the Primary Care Information System. Both datasets cover the period between 2016 and 2021, providing information on the number of municipalities, average number of diabetes cases in the population, hospitalizations due to diabetes-related complications and surgical treatments for diabetic foot in Ceará (6).

### Eligibility criteria 

The eligibility criteria for spatial analysis were established based on case records from the Primary Care Information System and the Hospital Information System, covering the period from 2016 to 2021. Inclusion criteria considered the total population of Ceará and records of people diagnosed with diabetes, diabetes-related hospitalizations and surgical treatment for diabetic foot, including records by municipality.

### Data organization and statistical analysis 

The annual incidence rate was calculated taking the number of cases of people with diabetes and diabetic foot surgery and the population diagnosed with diabetes in the state, multiplied by 10,000. For spatial analysis, incidence was standardized using the indirect method, using the mean year of the period (2018) as a reference (7). However, due to the heterogeneity of the rates and the instability of the values ​​between neighboring municipalities, they were smoothed using the local empirical Bayes method (8).

The choice of the local empirical Bayes smoothing method is justified by the fact that this method avoids the excessive smoothing observed in the full Bayes approach, while preserving relevant local variations, allowing identification of consistent regional patterns and areas with outliers of epidemiological interest (9-10). However, because it does not approximate municipal rates to a statewide (global) average, the results obtained should be interpreted with caution, as they may be subject to greater imprecision.

Thus, the application of this method brings the study closer to reality, considering not only data from a specific municipality, but also analyzing other municipalities neighboring it through a spatial proximity matrix (11). Spatial clusters were identified through a spatial autocorrelation function, to which the Local Moran’s Index (a Local Indicator of Spatial Association - LISA) was applied to verify the presence of spatial clusters and quantify the degree of spatial association in each municipality. This makes it possible to identify primary clusters, that is, those least likely to have occurred randomly (12).

This association is demonstrated in the Moran scatterplot, which identifies four quadrants: quadrant one (Q1) shows cities with high rates that are close to cities with equally high rates (high/high spatial pattern); quadrant two (Q2) shows cities with low rates that are surrounded by cities with equally low rates (low/low spatial pattern); quadrant three (Q3) shows cities with high rates that are surrounded by cities with low rates (high/low spatial pattern); finally, quadrant four (Q4) shows cities with low rates that are surrounded by cities with high rates (low/high spatial pattern), considering a p-value <0.05 (13).

Furthermore, the Getis-Ord Gi* technique was applied. This technique creates Z-scores for each municipality based on the desired indicator. Scores higher than the mean indicate high-incidence areas clustered with similar areas (hotspots), and Z-scores lower than the mean indicate low-incidence areas surrounded by similar areas (coldspots) (12). Finally, a purely spatial scanning analysis (focusing on the distribution and configuration of elements within a specific space) was performed to identify exclusively spatial clusters. To achieve this, the discrete Poisson model and the following rules were used: circular clusters, no cluster overlap, and a maximum cluster size of 999 replicates, as this is the number of replicates that ensures excellent analytical power for all types of datasets (14).

Relative risk (RR) was calculated for each municipality in Ceará, with those with RR>1 presenting a higher relative risk for the event of interest (12). The crude rate analysis, the local empirical Bayes analysis, the spatial autocorrelation test (Local Moran) calculation, and the Getis-Ord Gi* technique were performed using GeoDa 1.14 software. The purely spatial scan analysis was performed using SaTScan 9.7 software. All maps were produced using QGIS 3.16 software.

## Results 

Between 2016 and 2021, 7,319 diabetic foot surgery hospitalizations were recorded in Ceará. This total included debridement of devitalized tissue and amputations.

The Fortaleza macro-region stood out, with 49.8% (n=3,650) of hospitalizations. The same region also had the highest diabetes death rate (40.5%; n=5,501). Cases were predominant among males (51.6%; n=14,285), with the majority being of Brown (Brazilian mixed race) skin color (55.9%; n=15,463), and among older age groups, especially those aged 60 to 69 (26.2%; n=6,670).

The majority of the hospitalizations recorded did not specify the type of service that provided this care, whereby the majority of cases fell into the “unknown” category (83.4%; n=10,699). Therefore, it was not possible to determine whether care was provided in private or public services. Furthermore, 84.9% (n=2,440,091) of care referred to urgent cases (Table 1).

**Table 1:** Characterization of hospitalizations for surgical treatment of diabetic foot. Ceará, 2016-2021 (n=7,319)

Variables	n (%)
Sex	
Male	14,285 (51.6)
Female	13,361 (48.3)
**Hospitalizations by macro-region**	
Litoral Leste/Jaguaribe	323 (4.4)
Sertão Central	920 (12.5)
Cariri	1,241 (16.9)
Sobral	1,185 (16.1)
Fortaleza	3,650 (49.5)
**Race/skin color**	
White	2.630 (9.5)
Black	321 (1.1)
Brown	15.463 (55.9)
Asian	1,238 (4.4)
Indigenous	31 (0.1)
No information	7,963 (28.8)
**Age group** (years)	
20-29	770 (3.0)
30-39	1,233 (4.8)
40-49	2,739 (10.8)
50-59	5,033 (19.7)
60-69	6,670 (26.2)
70-79	5,526 (21.7)
≥80	3,467 (13.6)
**Hospitalization type**	
Public	1,521 (11.8)
Private	607 (4.7)
Unknown	10,699 (83.4)
**Nature of care**	
Elective	432,939 (15.0)
Urgent	2,440,091 (84.9)
**Deaths by macro-region**	
Litoral Leste/Jaguaribe	886 (6.5)
Sertão Central	1,195 (8.8)
Cariri	2,851 (21.0)
Sobral	3,135 (23.1)
Fortaleza	5,501 (40.5)

**Figure 2:**
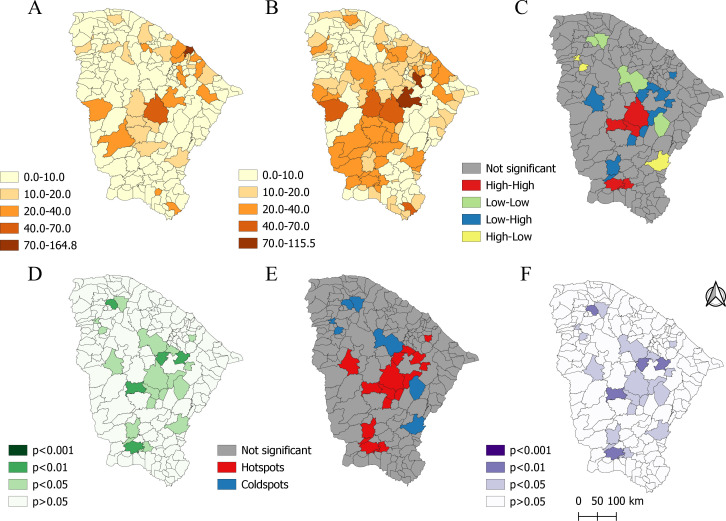
Spatial distribution of diabetic foot surgery performed in the state, crude rate per 100,000 inhabitants (A), after smoothing using the local empirical Bayes method (B), local Moran index (C), statistical significance (D), Getis-Ord Gi* (E), and statistical significance (F). Ceará, 2016-2021 (n=7,319)

The crude analysis of spatial distribution revealed a concentration of people undergoing surgical treatment for diabetic foot in Fortaleza (Figure 2A). After smoothing using the local empirical Bayes method (Figure 2B), a shift in the concentration of people undergoing treatment was seen, with municipalities such as Quixadá (Sertão Central) and Aracoiaba (metropolitan region of Fortaleza) presenting the highest rates.

The local Moran’s Index (Figure 2C) showed the regions that, after analysis, stood out as having high-high pattern municipalities, with two concentrations of diabetic foot surgical treatment cases visible. The first was located in the Central Sertão, between the municipalities of Quixeramobim, Pedra Branca and Senador Pompeu. The second was in the Cariri macro-region and encompassed three municipalities: Assaré Farias Brito, and Altaneira. These findings were corroborated by the statistical significance map (Figure 2D) in areas with a p-value <0.05. 

The Getis-Ord Gi* analysis (Figure 2E) showed hot spots in municipalities similar to those previously evidenced. However, other municipalities became added to the findings: Tamboril (Sobral region), Piquet Carneiro (Cariri region), Milhã, Banabuiú, Choró, Ibaretama, Ibicuitinga (Sertão Central region), Itapiúna and Ocara (metropolitan region of Fortaleza). All these municipalities presented a p-value <0.05 (Figure 2F).

**Figure 3:**
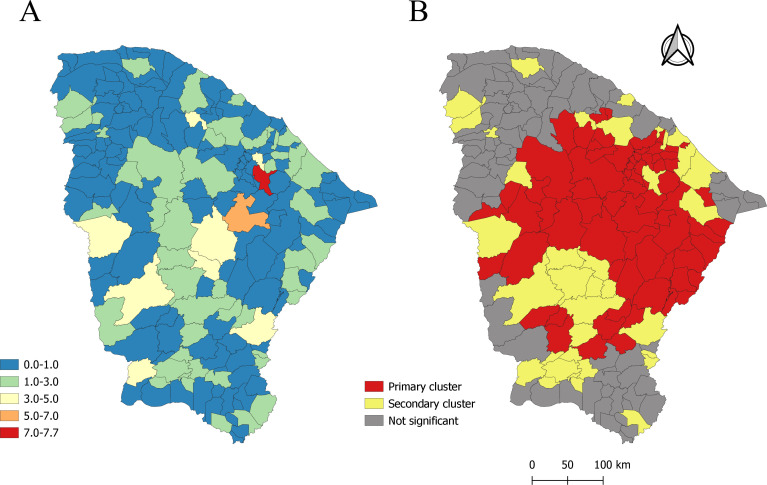
Spatial distribution of diabetic foot surgery performed in the state, purely spatial scanning for relative risk (A) and identification of primary and secondary clusters regarding the event of interest. Ceará, 2016-2021 (n=7,319)

Finally, the purely spatial scanning identified the municipalities with the highest relative risk of diabetic foot surgery (Figure 3A). In this analysis, the municipality that most stood out was Aracoiaba (metropolitan region of Fortaleza), followed by Quixadá (Sertão Central region). It is also noteworthy that 69.5% (n=128) of the state’s municipalities presented RR<1 (Figure 3A). Furthermore, another map (Figure 3B) was formulated to identify the primary cluster of the study, which included municipalities from all five macro-regions of the state.

## Discussion 

The results of this study highlighted the concentration of debridement and amputation cases arising from the extent and severity of diabetic foot lesions in Ceará. Factors associated with this situation include population density, greater availability of specialized services and possible regional disparities in access to health services between the state capital (Fortaleza) and the interior region of the state, particularly due to the spatial shift in the distribution of cases over the years, with municipalities in the interior emerging as new and significant areas of incidence of surgical treatment of diabetic foot.

It is important to highlight that, because this study is an analysis of secondary data from more than one health information system on foot complications in people with diabetes, it may have limitations. The Primary Care Information System may not incorporate information on care provided in specialized services, emergency rooms, hospitals or private healthcare services, thus limiting conclusions about the true extent of surgical treatment for foot complications in this population.

The predominance of this treatment in males corroborates a study (15) that estimated 9.2% prevalence of diabetes in males in Brazil, with regional variations of 6.3% in the North, 9.5% in the Northeast, 6.9% in the South, and 12.8% in the Southeast. Furthermore, lower limb ulcer prevalence is higher among certain population groups, such as Black, Latino and Native American people, as well as individuals in socioeconomically vulnerable situations (15-16). Cultural factors related to the male sex have historically attributed less importance to self-care in adulthood, favoring the emergence of complications and demands for surgical treatment of diabetic foot in this stage of life and in the first decade of old age. This contributes to increased economic costs for this population, compromising work and productive capacity, as well as quality of life (17).

Low adherence to self-care, combined with the low effectiveness of prevention services with protocols structured for this purpose, sometimes associated with the need for ongoing education of primary care health professionals on screening and early diagnosis of foot ulcers in people with diabetes, certainly contributes to the number of complex lesions and referral for care in specialized services or those with a higher level of complexity (18).

A study (19-20) showed that urgent care provision in healthcare services related to diabetic foot surgery resulted in approximately 43.7% of prolonged hospitalizations. This negatively impacts this sector due to the high costs of treatment, including the use of antibiotics in 83.1% of cases and the longer intensive treatment stay, with averages ranging from 8.4 days for patients with diabetes and foot complications to 5.6 days for those without such complications. A similar situation occurs regarding the mortality rate, which ranges from 231 deaths per 1,000 people per year among those with diabetes and foot complications to 182 per 1,000 people per year among those without (21).

Early screening for diabetic foot and regular monitoring of people with diabetes by trained health professionals, especially in primary care, including those with a history of previous amputation, depending on the level of risk of complications in the lower limbs, would certainly minimize case worsening and amputations (22), reducing these numbers.

In this context, underutilization of simple low-cost technologies in primary health care stands out, such as periodic diabetic foot assessments by healthcare professionals and monofilament sensitivity testing. Even the Ankle Brachial Index (ABI), a simple sonar test generally available in Primary Health Care Centers, can help determine the risk of foot complications in people with diabetes (23). Simple and, unfortunately, underutilized technologies, recognized in health care policies for people with chronic diseases in Brazil and worldwide, could be widely used to prevent health complications in people with diabetes.

Health promotion and prevention of surgical treatment of diabetic foot require blood glucose level monitoring, continuous assessment with guidance on foot care and availability of specialized diabetes centers (20,22).

These facilities should have a multidisciplinary team consisting of an endocrinologist, angiologist, stoma care nurse, physiotherapist, nutritionist, psychologists and social workers, in addition to providing orthopedic footwear and special insoles to prevent foot ulcers (22). Such measures are essential to reduce the demand for urgent care in health services, as well as the number of recurrent amputations.

Incorporation of and investment in healthcare technologies has also been encouraged, particularly for enhancing early identification of diabetic foot complications and increasing treatment adherence. These technologies include innovative technologies such as smart sensors in insoles, body pressure and temperature monitoring socks, artificial intelligence for predictive analysis and imaging diagnostics, as well as telemedicine and mobile apps for remote monitoring (22).

However, this is not the reality for most health services, as many municipalities in interior regions do not offer specialized services or care to people with diabetes, highlighting that inequality in access to health services can also be a determining factor in the spatial distribution of diabetic foot complications.

Spatial analysis corroborates this statement by revealing the problem of significant clusters in specific regions of Ceará, such as the Sertão Central and the Cariri macro-regions, both with Human Development Indices (HDI) below 0.659, with a large proportion of revenue coming from external sources, highlighting economic and social vulnerability (6). This pattern has been described previously in an ecological study conducted in Northeast Brazil (24), in which the authors demonstrated that areas with lower HDI had a higher concentration of diabetic foot surgery hospitalizations.

As indicated in other studies (25), identified clusters possibly face significant challenges related to the management of diabetes complications, such as prevention, screening and early identification of diabetic foot complications by primary health care services. They also face difficulties in the provision of care slots, patient access to specialized services, treatment of diabetic foot complications, adherence to treatment by those who need it, and low investment in healthcare (26). These realities certainly influence the number of diabetic foot surgical treatments.

The data obtained in this study, in turn, highlight the socioeconomic disparity between municipalities in the interior of Ceará as influencing cases requiring diabetic foot surgical treatment. Therefore, we suggest further investigation into local and regional aspects, particularly in the clusters identified, in order to explain the spatial distribution of cases.

Results like those of this study become relevant in the development of government strategies in these regions, thanks to their potential contribution to strategic health planning. Such management is essential for promoting equity in access to health services, encouraging preventive practices and improving infrastructure in interior-region cities, reducing regional inequalities and improving the population’s quality of life.
