# Supplementary material for: Surgical treatment of diabetic foot: a spatial analysis, Ceará, Brazil, 2016-2021
Source: Epidemiol Serv Saude. 2025 Dec 19;35:e20240755. doi: 10.1590/S2237-96222026v35e20240755.en (PMC12716125; doi:10.1590/S2237-96222026v35e20240755.en)
Supplement: Translation

# Translation: Tratamento cirúrgico do pé diabético: análise espacial, Ceará, 2016-2021

**DOI:** 10.1590/S2237-96222026v35e20240755.pt

**Authors:** Yterfania Soares Feitosa, Bruno Victor Barros Cabral, Gabriela de Sousa Lima, Emiliana Bezerra Gomes, George Jó Bezerra Sousa, Thereza Maria Magalhães Moreira, Maria Lúcia Duarte Pereira

**Affiliations:** 1 Universidade Estadual do Ceará, Programa de Pós-Graduação em Cuidados Clínicos em Enfermagem e Saúde, Fortaleza, CE, Brasil; 2 Universidade Regional do Cariri, Enfermagem, Crato, CE, Brasil; 3 Ministério da Saúde, Brasília, DF, Brasil

**Keywords:** Diabetes, Pé Diabético, Amputação Cirúrgica, Desbridamento, Estudos Observacionais

## Abstract

**Objetivo:**

Identificar o padrão espacial do tratamento cirúrgico do pé diabético com complicações no Ceará.

**Métodos:**

Estudo observacional de análise espacial realizado no Ceará. Para o estudo, calculou-se a taxa de incidência anual do tratamento cirúrgico do pé diabético. Essa taxa foi padronizada pelo método indireto usando o ano médio (2018). Posteriormente, a taxa foi suavizada pelo método bayesiano empírico local. Além disso, foi aplicada a técnica Getis-Ord Gi* para indicar áreas de alta incidência agrupadas. Por fim, realizou-se a varredura puramente espacial para identificar o risco relativo (RR) para cada município do Ceará, sendo que aqueles com RR>1 apresentam maior risco para o evento de interesse.

**Resultados:**

Entre 2016 e 2021, foram registradas 7.319 hospitalizações para tratamento cirúrgico do pé diabético complicado no Ceará, com destaque para a macrorregião de Fortaleza (49,8%; n=3.650). Na análise espacial, as taxas brutas, bem como após suavização bayesiana empírica local, evidenciam maior concentração nas seguintes macrorregiões: Fortaleza, Sertão Central, Sobral e Cariri. Essa informação é confirmada pelo Moran local e Getis-Ord Gi* (p-valor <0,05). Na varredura, destacou-se que 30,5% (n=56) dos municípios do estado apresentaram RR>1.

**Conclusão:**

O estudo evidenciou um padrão espacial marcado pela concentração de casos de tratamento cirúrgico do pé diabético na macrorregião de Fortaleza e, após a suavização bayesiana, desvelou o deslocamento na distribuição dos casos para dois municípios do interior, Quixadá e Aracoiaba.

**Aspectos éticos**

Esta pesquisa utilizou bancos de dados de domínio público e anonimizados.

## Introdução 

Até 2045, a prevalência do diabetes tipo 2 deve aumentar 21,1% em países subdesenvolvidos, 12,2% em países desenvolvidos e 11,9% em países de baixo desenvolvimento (1). Dentre as complicações associadas à doença, destacam-se as úlceras nos pés, que são especialmente preocupantes, devido ao impacto significativo na morbidade e na mortalidade, além de gerar incapacidades (2). 

As complicações graves associadas ao diabetes, como as úlceras podálicas, quando não diagnosticadas e tratadas precocemente, podem evoluir para infecções e amputações de membros inferiores. Tais complicações aumentam as taxas de mortalidade e resultam em incapacidade física, redução da atividade laboral, aumento dos custos para os serviços de saúde e danos sociais e emocionais (2). 

Em 2022, no Brasil, foram registrados 18.811 casos de amputações, dos quais 952 ocorreram no Ceará, representando um aumento de 244,9% em relação a 2013. No ranking nacional, o Ceará ocupou a 17ª posição e a terceira posição no Nordeste, com uma taxa de 10,9 amputações por 100 mil habitantes. A região do Cariri, no Ceará, apresentou a taxa mais elevada, 13,7 amputações por 100 mil habitantes (3). 

Nesse contexto, a análise da distribuição espacial das amputações de membros inferiores é uma estratégia crucial para compreender as variações geográficas e identificar áreas de alta prevalência. A autocorrelação delas permite detectar padrões geográficos significativos que podem indicar a concentração de casos em determinadas regiões, com capacidade de direcionar o planejamento de ações em saúde. 

A exploração desses padrões espaciais é essencial para desvendar fatores subjacentes à concentração de casos nos territórios em relação ao tempo e identificar possíveis causas ou determinantes sociais, como desigualdades socioeconômicas, barreiras no acesso aos serviços de saúde e variáveis ambientais que influenciam na ocorrência dessas amputações (4). 

Portanto, este estudo demonstra sua relevância ao identificar as áreas geográficas mais afetadas no estado do Ceará, permitindo uma análise detalhada dos dados sobre o tratamento cirúrgico do pé diabético, com potencial de subsidiar gestores e profissionais de saúde na elaboração de estratégias de intervenção preventiva dessas ocorrências. 

Pensar ações que otimizem a alocação de recursos para atos de promoção da saúde e de prevenção de agravos às regiões com maior número de amputações certamente impactaria na qualidade de vida de pessoas com diabetes e complicações podálicas, bem como reduziria os custos sociais e econômicos dessas pessoas e do Sistema Único de Saúde. Assim, o objetivo do estudo é identificar o padrão espacial do tratamento cirúrgico do pé diabético no Ceará, Brasil.

## Métodos 

### Desenho do estudo 

Trata-se de um estudo observacional de análise espacial realizado no Ceará que utilizou como unidade de análise todos os 184 municípios que compõem o estado. O estudo seguiu as recomendações do Strengthening the Reporting of Observational Studies in Epidemiology (STROBE) (5). 

### Contexto 

O Ceará é dividido em cinco macrorregiões de saúde: Litoral Leste, Sertão Central, Cariri, Sobral e Fortaleza, com uma extensão territorial de 148.894,447 km^2^ e população estimada de 8.794.957 pessoas (Figura 1). Este estudo utilizou dados do Sistema de Informação Hospitalar, acessados pelo Departamento de Informática do Sistema Único de Saúde, referentes aos casos de tratamento cirúrgico do pé diabético (revascularização, desbridamento e amputação) (3) relacionado às complicações de membros inferiores em pessoas com diabetes (6). 

**Figura 1:** Representação geográfica do estado no Brasil (A) e sua divisão em cinco macrorregiões de saúde (B). Ceará, 2025

Além disso, foram consideradas informações sobre a população com diabetes no estado, registradas no Sistema de Informação para Atenção Básica. Ambos os conjuntos de dados compreendem o período entre 2016 e 2021, abrangendo informações sobre número de municípios, média de casos de diabetes na população, hospitalizações por complicações relacionadas à diabetes e tratamentos cirúrgicos por pé diabético no Ceará (6). 

### Critérios de elegibilidade 

Os critérios de elegibilidade para a análise espacial foram estabelecidos com base nos registros de casos do Sistema de Informação para Atenção Básica e no Sistema de Informação Hospitalar, abrangendo o período de 2016 a 2021. Os critérios de inclusão consideraram a população total do Ceará e os registros de pessoas diagnosticadas com diabetes, hospitalizações relacionadas ao diabetes e tratamentos cirúrgicos por pé diabético, incluindo os registros por município.

### Organização e análise estatística dos dados 

A taxa de incidência anual foi calculada utilizando-se o número de casos de pessoas com diabetes e tratamento cirúrgico do pé diabético pela população com diagnóstico de diabetes no estado multiplicado por 10 mil. Para a análise espacial, a incidência foi padronizada pelo método indireto, aplicando-se como referência o ano médio do período (2018) (7). No entanto, devido à heterogeneidade das taxas e à instabilidade dos valores entre municípios vizinhos, elas foram suavizadas utilizando o método bayesiano empírico local (8).

A escolha pelo método de suavização bayesiano empírico local se justifica pelo fato de que esse método evita o abrandamento excessivo observado na abordagem totalmente bayesiana, ao mesmo tempo em que preserva variações locais relevantes, permitindo a identificação de padrões regionais consistentes e de áreas com valores atípicos de interesse epidemiológico (9-10). Contudo, por não realizar a aproximação das taxas municipais a uma média estadual (global), os resultados obtidos devem ser interpretados com cautela, uma vez que podem estar sujeitos a maior imprecisão.

Assim, a aplicação desse método aproxima o estudo da realidade, levando em consideração não somente os dados de um município específico, mas também analisando os demais municípios que o limitam através de uma matriz de proximidade espacial (11). Os aglomerados espaciais foram identificados por meio de uma função de autocorrelação espacial, na qual foi aplicado o Índice Moran Local (LISA) para verificar a presença de aglomerados espaciais e quantificar o grau de associação espacial em cada município. Dessa forma, é possível identificar aglomerados primários, ou seja, aqueles com menor probabilidade de terem ocorrido aleatoriamente (12).

Essa associação é demonstrada no diagrama de dispersão de Moran, que identifica quatro quadrantes: o quadrante um (Q1) mostra cidades com índices elevados e que estão próximas a cidades com índices igualmente altos (padrão espacial alto/alto); o quadrante dois (Q2) mostra cidades com índices baixos que são cercadas por cidades com índices igualmente baixos (padrão espacial baixo/baixo); no quadrante três (Q3) estão cidades com índices elevados e que são cercadas por cidades com índices baixos (padrão espacial alto/baixo); por fim, no quadrante quatro (Q4) estão cidades com baixos índices que são cercadas por cidades com índices altos (padrão espacial baixo/alto), considerando p-valor<0,05 (13). 

Além disso, foi aplicada a técnica Getis-Ord Gi*. A técnica cria escores Z para cada município com base no indicador desejado. Escores maiores que a média indicam áreas de alta incidência agrupadas com áreas de incidência semelhante (áreas quentes), e os escores Z menores que a média indicam áreas de baixa incidência cercadas por áreas semelhantes (áreas frias) (12). Ao final, foi realizada uma análise de varredura puramente espacial (aquela que se concentra na distribuição e na configuração de elementos dentro de um espaço específico) que objetivou identificar agrupamentos exclusivamente espaciais. Para isso, utilizou-se o modelo de Poisson discreto e as seguintes regras: agrupamentos circulares, ausência de sobreposição de agrupamentos, tamanho máximo de agrupamento igual a 999 replicações, uma vez que esse é o número de replicações que garante a excelência no poder de análise para todos os tipos de conjuntos de dados (14). 

O risco relativo (RR) foi calculado para cada município do Ceará, sendo que aqueles com RR>1 apresentam maior risco relativo para o evento de interesse (12). A análise da taxa bruta, a bayesiana empírica local, o cálculo do teste de autocorrelação espacial (Moran Local) e a técnica Getis-Ord Gi* foram realizadas no software GeoDa 1.14. A análise de varredura puramente espacial foi realizada no software SaTScan 9.7. Todos os mapas foram produzidos no software QGIS 3.16. 

## Resultados 

No Ceará, entre 2016 e 2021, foram registradas 7.319 hospitalizações para casos de tratamentos cirúrgicos do pé diabético. Na soma, incluíram-se o desbridamento de tecidos desvitalizados e a realização de amputações.

A macrorregião de Fortaleza se destacou, com 49,8% (n=3.650) das hospitalizações. Na mesma região, também se observou a maior taxa de óbitos (40,5%; n=5.501) por diabetes. Predominou na população o sexo masculino (51,6%; n=14.285), com maioria parda (55,9%; n=15.463) e em faixas etárias mais avançadas, especialmente entre 60 e 69 anos (26,2%; n=6.670). 

A maioria das hospitalizações registradas não possuía o regime especificado do tipo de serviço que prestou o atendimento, sendo a maior parte dos casos considerados “ignorados” (83,4%; n=10.699). Dessa forma, não foi possível determinar se o atendimento ocorreu em serviços privados ou públicos. Além disso, 84,9% (n=2.440.091) dos atendimentos foram de urgência (Tabela 1). 

**Tabela 1:** Caracterização das internações para tratamento cirúrgico do pé diabético. Ceará, 2016-2021 (n=7.319)

Variáveis	n (%)
Sexo	
Masculino	14.285 (51,6)
Feminino	13.361 (48,3)
**Internações por macrorregião**	
Litoral Leste/Jaguaribe	323 (4,4)
Sertão Central	920 (12,5)
Cariri	1.241 (16,9)
Sobral	1.185 (16,1)
Fortaleza	3.650 (49,5)
**Raça/cor da pele**	
Branca	2.630 (9,5)
Preta	321 (1,1)
Parda	15.463 (55,9)
Amarela	1.238 (4,4)
Indígena	31 (0,1)
Sem Informação	7.963 (28,8)
**Faixa etária (anos)**	
20-29	770 (3,0)
30-39	1.233 (4,8)
40-49	2.739 (10,8)
50-59	5.033 (19,7)
60-69	6.670 (26,2)
70-79	5.526 (21,7)
≥80	3.467 (13,6)
**Regime de internação**	
Público	1.521 (11,8)
Privado	607 (4,7)
Ignorado	10.699 (83,4)
**Caráter de atendimento**	
Eletivo	432.939 (15,0)
Urgência	2.440.091 (84,9)
Óbitos	
Litoral Leste/Jaguaribe	886 (6,5)
Sertão Central	1.195 (8,8)
Cariri	2.851 (21,0)
Sobral	3.135 (23,1)
Fortaleza	5.501 (40,5)

A investigação quanto à distribuição espacial evidenciou uma concentração bruta de pessoas em tratamentos cirúrgicos do pé diabético em Fortaleza (Figura 2A). Após suavização pelo método bayesiano empírico local (Figura 2B), observou-se um deslocamento da concentração de pessoas em tratamento, com municípios como Quixadá (Sertão Central) e Aracoiaba (região metropolitana de Fortaleza) apresentando as maiores taxas. 

**Figura 2:** Distribuição espacial no tratamento cirúrgico do pé diabético realizado no estado, taxa bruta por 100 mil habitantes (A), após suavização pelo método bayesiano empírico local (B), índice Moran local (C), significância estatística (D), Getis-Ord Gi* (E), e significância estatística (F). Ceará, 2016-2021 (n=7.319)

O Índice de Moran local (Figura 2C) apresentou as regiões que, após análise, destacaram-se com municípios de padrão alto-alto, sendo visíveis duas concentrações de casos de tratamento cirúrgico do pé diabético. A primeira se localizou no Sertão Central, entre os municípios de Quixeramobim, Pedra Branca e Senador Pompeu. A segunda, está na macrorregião do Cariri e engloba três municípios: Assaré, Farias Brito e Altaneira. Tais achados foram corroborados pelo mapa de significância estatística (Figura 2D) em áreas com p-valor<0,05. 

A análise pelo Getis-Ord Gi* (Figura 2E) apresentou áreas quentes em municípios semelhantes aos já evidenciados anteriormente. Entretanto, outros municípios se somaram aos achados: Tamboril (região Sobral), Piquet Carneiro (região Cariri), Milhã, Banabuiú, Choró, Ibaretama, Ibicuitinga (região Sertão Central), Itapiúna e Ocara (região metropolitana de Fortaleza). Todos esses municípios apresentaram p-valor<0,05 (Figura 2F). 

Por fim, a varredura puramente espacial identificou os municípios com maior risco relativo de tratamento cirúrgico do pé diabético (Figura 3A). Nessa análise, o município em destaque foi Aracoiaba (região metropolitana de Fortaleza), seguido por Quixadá (região Sertão Central). Ressalta-se ainda que 69,5% (n=128) dos municípios do estado apresentaram RR<1 (Figura 3A). Ademais, outro mapa (Figura 3B) foi formulado para identificação do aglomerado primário do estudo, que incluiu municípios de todas as cinco macrorregiões do estado. 

**Figura 3:** Distribuição espacial do tratamento cirúrgico do pé diabético realizado no estado, varredura puramente espacial para risco relativo (A) e identificação de aglomerados primários e secundários quanto ao evento de interesse. Ceará, 2016-2021 (n=7.319)

## Discussão 

Os resultados deste estudo evidenciaram a concentração dos casos de desbridamento e de amputações decorrentes de extensão e de gravidade das lesões do pé diabético no Ceará. Como fatores associados a essa realidade, sugere-se a influência de fatores como densidade populacional, maior oferta de serviços especializados e possíveis disparidades regionais no acesso ao serviço de saúde entre a capital e o interior, especialmente pelo deslocamento espacial ao longo dos anos na distribuição dos casos, com os municípios do interior emergindo com novas e significativas áreas de incidência no tratamento cirúrgico do pé diabético.

É importante destacar que, por se tratar de uma análise com dados secundários e oriundos de mais de um sistema de informação em saúde sobre as complicações nos pés de pessoas com diabetes, este estudo pode apresentar limitações. O Sistema de Informação para Atenção Básica pode não incorporar informações de atendimentos nos serviços especializados, nas emergências, nos hospitais ou naqueles da rede privada, restringindo, assim, as conclusões sobre a real magnitude do tratamento cirúrgico de complicações podálicas na população. 

A predominância desse tratamento em pessoas do sexo masculino corrobora um estudo (15) que estima, para o Brasil, uma prevalência de 9,2% do diabetes em homens, com variações regionais de 6,3% no Norte, 9,5% no Nordeste, 6,9% no Sul e 12,8% no Sudeste. Além disso, determinados grupos populacionais, como negros, hispânicos, nativos americanos e indivíduos em situação de vulnerabilidade socioeconômica, apresentam maior prevalência de úlceras nos membros inferiores (15-16). Fatores culturais relacionados ao sexo masculino, historicamente, atribuem a eles menos importância ao autocuidado na vida adulta, favorecendo o surgimento de complicações e de demandas pelo tratamento cirúrgico do pé diabético nessa fase e na primeira década da fase idosa, o que contribui para aumento dos custos econômicos dessa população, comprometendo a capacidade laboral e produtiva, bem como a qualidade de vida (17). 

A baixa adesão ao autocuidado, aliada à baixa efetividade dos serviços de prevenção com protocolos estruturados para este fim, por vezes associada à necessidade de educação permanente dos profissionais de saúde da atenção primária sobre o rastreio e o diagnóstico precoce de úlceras nos pés de pessoas com diabetes, certamente contribui para o número de lesões complexas e para a busca por atendimento em serviços especializados ou com maior nível de complexidade (18). 

Um estudo (19-20) evidenciou-se que os atendimentos de urgência nos serviços de saúde relacionados ao tratamento cirúrgico do pé diabético resultaram em aproximadamente 43,7% dos casos de hospitalizações prolongadas. O que causa um impacto negativo nesse setor devido aos elevados custos do tratamento, incluindo o uso de antibióticos em 83,1% dos casos e o tratamento intensivo de maior permanência, com médias variando entre 8,4 dias, para os pacientes com diabetes e com complicações nos pés, e 5,6 dias, para aqueles sem tais complicações. Situação semelhante ocorre na taxa de mortalidade, que varia entre 231 óbitos por 1 mil pessoas/ano, entre pessoas com diabetes e com complicações nos pés, e 182 por 1 mil pessoas/ano, entre aquelas que não as têm (21). 

O rastreamento precoce do pé diabético e o acompanhamento regular de pessoas com diabetes por profissionais de saúde capacitados, sobretudo na atenção primária, incluindo nesse cuidado aquelas com histórico de amputação prévia, conforme o nível de risco de complicações em membros inferiores, certamente minimizaria agravamentos e amputações (22), reduzindo esses números.

Nesse contexto, destaca-se a subutilização de tecnologias simples e de baixo custo na atenção primária de saúde, como a avaliação periódica do pé diabético pelo profissional de saúde e a realização do teste de sensibilidade com monofilamento. Até mesmo o Índice Tornozelo Braquial (ITB), exame simples realizado com um sonar e geralmente disponível nas Unidades Básicas de Saúde, pode colaborar na determinação do risco de complicações nos pés de pessoas com diabetes (23). Tecnologias simples e, infelizmente, mal aproveitadas, reconhecidas na política de atenção às pessoas com doenças crônicas no Brasil e no mundo, poderiam ser utilizadas largamente na prevenção de complicações na saúde de pessoas com diabetes.

A promoção da saúde e a prevenção do tratamento cirúrgico do pé diabético requerem monitorização dos níveis glicêmicos, avaliação contínua com orientação sobre os cuidados com os pés e disponibilização de centros especializados em diabetes (20,22). 

Esses locais devem contar com uma equipe multidisciplinar composta por endocrinologista, angiologista, enfermeiro estomaterapeuta, fisioterapeuta, nutricionista, psicólogos e profissionais do serviço social, além de oferecer calçados ortopédicos e palmilhas especiais para prevenção de úlceras nos pés (22). Tais medidas são essenciais para reduzir a demanda por atendimentos de urgência nos serviços de saúde, bem como o número de amputações recorrentes.

A incorporação e o investimento em tecnologias para o cuidado em saúde também têm sido estimulados, especialmente por potencializar a identificação precoce de complicações no pé diabético e o aumento na adesão ao tratamento. Dentre elas, destacam-se tecnologias inovadoras, como sensores inteligentes em palmilhas, meias de monitoramento da pressão e da temperatura corporal, inteligência artificial para análise preditiva e diagnóstico por imagem, além de telemedicina e de aplicativos móveis para acompanhamento remoto (22). 

No entanto, essa não é a realidade da maioria dos serviços de saúde, visto que muitos municípios interioranos não oferecem um serviço ou uma assistência especializada às pessoas com diabetes, reforçando que a desigualdade no acesso aos serviços de saúde também pode ser um fator determinante na distribuição espacial das complicações do pé diabético.

A análise espacial reforça essa ideia ao desvelar a problemática de aglomerados significativos em regiões específicas do Ceará, como o Sertão Central e a macrorregião do Cariri, ambas com Índice de Desenvolvimento Humano (IDH) abaixo de 0,659, com grande parte da receita vinda de fontes externas, evidenciando vulnerabilidade econômica e social (6). Esse padrão foi descrito anteriormente em um estudo ecológico realizado no Nordeste brasileiro (24), no qual os autores demonstraram que áreas com menor IDH apresentavam maior concentração de hospitalizações para tratamento cirúrgico do pé diabético. 

Como apontado em outros estudos (25), os aglomerados identificados, possivelmente, enfrentam desafios significativos relacionados ao manejo das complicações do diabetes, como prevenção, rastreamento e identificação precoce de complicações do pé diabético pela atenção primária de saúde. Além de se depararem com dificuldades na oferta de vagas de atendimento, no acesso dos pacientes a serviços especializados, no tratamento de complicações do pé diabético, na adesão dos necessitados ao tratamento e, ainda, com baixo investimento em saúde (26). Realidades que certamente influenciam o quantitativo de tratamentos cirúrgicos para o pé diabético.

Os dados obtidos neste estudo, por sua vez, evidenciam a disparidade socioeconômica entre os municípios do interior do Ceará como influenciador dos casos de necessidade de tratamento cirúrgico do pé diabético. Assim, sugere-se a realização de investigações mais aprofundadas sobre aspectos locais e regionais, sobretudo nos aglomerados encontrados, que se proponham a explicar a distribuição espacial dos casos.

Resultados como os deste estudo tornam-se relevantes no desenvolvimento de estratégias governamentais nessas regiões, graças à potencial contribuição para o planejamento estratégico em saúde. Gestão essa que se mostra essencial para promover a equidade no acesso aos serviços de saúde, incentivar práticas preventivas e incrementar a infraestrutura nas cidades interioranas, reduzindo as desigualdades regionais e melhorando a qualidade de vida da população.
